# ASSOCIATION BETWEEN LIMITED ENGLISH PROFICIENCY AND MEDICARE ENROLLMENT CHOICE

**DOI:** 10.1093/geroni/igac059.2922

**Published:** 2022-12-20

**Authors:** Lilly Estenson, Mireille Jacobson

**Affiliations:** University of Southern California, Los Angeles, California, United States; University of Southern California, Los Angeles, California, United States

## Abstract

Choosing between traditional Medicare and Medicare Advantage (MA) is an important health and financial decision that can be especially complicated for Medicare beneficiaries who have limited proficiency speaking or reading English (LEP). In this study, we used data from the 2016–2018 Medicare Current Beneficiary Survey and ordinary least squares regression to examine the association between LEP and MA enrollment. Among the 19,621 respondents (32,912 person-year observations) who answered LEP questions, 9.2% self-reported having LEP and 38% were enrolled in MA. Limited proficiency reading English was significantly associated with enrollment choice; respondents who reported reading English “not well” or “not at all” were 6.7 percentage points less likely to enroll in MA than respondents who reported reading English “well” or “very well” (SE=0.025; p < 0.01). However, when we stratified respondents by language spoken at home, we discovered considerable variation in this association among language subgroups (English: -6.5 percentage points, SE=0.029, p < 0.05; Spanish: -1.5 percentage points, SE=0.051, p=NS; other: -11.2 percentage points, SE=0.065, p < 0.10). Additionally, respondents with limited proficiency reading English were 8.0 percentage points less likely to positively rate their Medicare knowledge (SE=0.022, p < 0.01) and 6.4 percentage points less likely to review their Medicare options annually (SE=0.023, p < 0.01) than respondents with English reading proficiency. Limited proficiency speaking English was not significantly associated with MA enrollment, Medicare knowledge, or annual options review. These findings suggest that English literacy rather than speaking proficiency may be a crucial determinant of enrollment choices. Language access around health insurance information is critical for equitable Medicare enrollment.

